# Rationale and Protocol for a Randomized Controlled Trial Comparing Fast versus Slow Weight Loss in Postmenopausal Women with Obesity—The TEMPO Diet Trial

**DOI:** 10.3390/healthcare6030085

**Published:** 2018-07-20

**Authors:** Radhika V. Seimon, Alice A. Gibson, Claudia Harper, Shelley E. Keating, Nathan A. Johnson, Felipe Q. da Luz, Hamish A. Fernando, Michael R. Skilton, Tania P. Markovic, Ian D. Caterson, Phillipa Hay, Nuala M. Byrne, Amanda Sainsbury

**Affiliations:** 1 Faculty of Medicine and Health, Charles Perkins Centre, The University of Sydney, the Boden Institute of Obesity, Nutrition, Exercise & Eating Disorders, Camperdown, NSW 2006, Australia; radhika.seimon@sydney.edu.au (R.V.S.); alice.gibson@sydney.edu.au (A.A.G.); claudia.harper@sydney.edu.au (C.H.); nathan.johnson@sydney.edu.au (N.A.J.); felipe.quintodaluz@sydney.edu.au (F.Q.d.L.); hamish.fernando@sydney.edu.au (H.A.F.); michael.skilton@sydney.edu.au (M.R.S.); tania.markovic@sydney.edu.au (T.P.M.); ian.caterson@sydney.edu.au (I.D.C.); 2 School of Human Movement and Nutrition Sciences, Centre for Research on Exercise, Physical Activity and Health, The University of Queensland, Brisbane, QLD 4072, Australia; s.keating@uq.edu.au; 3 Faculty of Health Sciences, The University of Sydney, Lidcombe, NSW 2141, Australia; 4 Faculty of Science, School of Psychology, The University of Sydney, Camperdown, NSW 2006, Australia; 5 Metabolism & Obesity Services, Royal Prince Alfred Hospital, Camperdown, NSW 2050, Australia; 6 School of Medicine, Western Sydney University, Translational Health Research Institute (THRI), Locked Bag 1797, Penrith, NSW 2751, Australia; P.Hay@westernsydney.edu.au; 7 School of Health Sciences, College of Health and Medicine, University of Tasmania, Launceston, TAS 7250, Australia; nuala.byrne@utas.edu.au

**Keywords:** weight loss, diet-reducing, obesity, clinical protocol, rationale

## Abstract

Very low energy diets (VLEDs), commonly achieved by replacing all food with meal replacement products and which result in fast weight loss, are the most effective dietary obesity treatment available. VLEDs are also cheaper to administer than conventional, food-based diets, which result in slow weight loss. Despite being effective and affordable, these diets are underutilized by healthcare professionals, possibly due to concerns about potential adverse effects on body composition and eating disorder behaviors. This paper describes the rationale and detailed protocol for the TEMPO Diet Trial (**T**ype of **E**nergy **M**anipulation for **P**romoting optimal metabolic health and body composition in **O**besity), in a randomized controlled trial comparing the long-term (3-year) effects of fast versus slow weight loss. One hundred and one post-menopausal women aged 45–65 years with a body mass index of 30–40 kg/m^2^ were randomized to either: (1) 16 weeks of fast weight loss, achieved by a total meal replacement diet, followed by slow weight loss (as for the SLOW intervention) for the remaining time up until 52 weeks (“FAST” intervention), or (2) 52 weeks of slow weight loss, achieved by a conventional, food-based diet (“SLOW” intervention). Parameters of body composition, cardiometabolic health, eating disorder behaviors and psychology, and adaptive responses to energy restriction were measured throughout the 3-year trial.

## 1. Introduction

The worldwide prevalence of obesity is increasing at an alarming rate [1]. Obesity is associated with a number of complications including type 2 diabetes, cardiovascular disease as well as many cancers [2], and is responsible for significant health care costs [1,3]. In Australia, in 2008 alone, overweight and obesity cost Australian society and government AU$56.6 billion in direct and indirect costs [4], while in the USA the direct medical costs of obesity were estimated at US$190 billion in 2012 [5], with the annual costs of treating obesity-related diseases in the USA estimated to increase by an additional US$48–$66 billion by 2030 [6]. However, if obesity were effectively treated, the costs of obesity-related health complications would be significantly reduced [4]. Thus, implementing effective obesity treatments is essential to reducing obesity-related comorbidities and the associated costs.

Management strategies for weight reduction include lifestyle interventions (i.e., changes in diet, physical activity and behavior), pharmacological treatment [7] and surgery [8,9]. However, lifestyle interventions are considered the first-line treatment for overweight and obesity, and are also an important component of pharmacological and surgical treatments [10]. Recent studies have noted that weight loss of as little as 3–5% of initial body weight can induce clinically meaningful reductions in some cardiovascular risk factors, diabetes and osteoarthritis, with larger weight losses producing even greater health benefits [10,11,12,13]. Although weight loss can usually be achieved through lifestyle interventions, the overwhelming majority of people regain the weight lost over the long-term (several years). A major reason for this weight regain is that the body responds to energy restriction and weight loss with a series of adaptive responses such as an increased drive to eat, a decrease in physical activity, and a decrease in energy expenditure [14,15]. In addition, energy restriction and weight loss induce adaptive responses in neuroendocrine status that may have adverse consequences on body composition, such as loss of bone mineral density (BMD) and lean mass, as well as muscle strength (which together potentially increase the risk of osteoporosis, sarcopenia and frailty), and promote regain of visceral adipose tissue (VAT), in turn increasing the risk of cardiometabolic diseases such as type 2 diabetes and cardiovascular disease [15].

As mentioned, greater weight losses in people with overweight or obesity are usually associated with greater health benefits. Although a number of dietary approaches have been advocated for the treatment of obesity [16,17], the dietary interventions that induce the greatest and longest-lasting weight losses are very low energy diets (VLEDs) [18]. These, by definition, involve restricting dietary energy intake to <3350 kJ (<800 kcal) per day [19]. VLEDs are most commonly administered as total meal replacement diets, which involve replacing all, or almost all, foods with nutritionally replete meal replacement products such as shakes, soups, bars, or desserts. The short-term success of VLEDs is high, resulting in fast and substantial weight losses of ~1.5–2.5 kg per week, typically over periods of 8–16 weeks [20], while reducing hunger and increasing satiety [21]. An individual commencing a VLED has an ~80% chance of losing ≥12.5% of their initial body weight, compared to ~50% of people commencing conventional, food-based diets that involve moderately restricting dietary energy intake by ~2000 kJ (500 kcal) per day, inducing slow weight loss [22]. The long-term success of VLEDs may also be greater than conventional, food-based diets, because greater initial weight loss has been shown to be predictive of long-term success in weight maintenance [23,24]. This is in keeping with recent evidence showing that there is no faster weight regain in response to fast than to slow weight loss [22,25,26]. Moreover, at 1 and 5 years after commencement of a VLED, ~60% and ~30% of people weigh ≥10% less than their initial body weight, respectively [18,27]. In addition, a meta-analysis showed that weight loss at 4–5 years after a VLED or a conventional, food-based diet was 6.6% versus 2.1%, respectively [18]. Besides achieving greater long-term weight loss, VLEDs are approximately three times cheaper to administer than conventional, food-based diets, in terms of the cost of dietetics support [28]. In terms of the cost to consumers, total meal replacement products—when used in place of all meals and snacks—are cheaper than the average per capita food expenditure [29].

Despite being an effective and affordable option for the dietary treatment of obesity, VLEDs are underutilized by healthcare professionals [28,30]. A 2002 survey of dietitians in Australia revealed that only 3.2% of respondents reported prescribing a VLED or fast weight loss to manage overweight or obesity in their clients [30]. This was corroborated in a 2008 survey, which revealed that only 1.5% of dietitians in Australia recommended VLEDs to their clients for weight loss [28]. Part of the reason for this underutilization of VLEDs may be due to concerns regarding adverse psychological outcomes, particularly with regards to inducing or exacerbating binge eating disorders, although these concerns may be unfounded [31]. Other safety concerns are the possibility that fast weight loss may have adverse effects on body composition. Indeed, the greater degree of energy restriction used to achieve fast weight loss could conceivably induce stronger adaptive responses than those seen during slow weight loss with a conventional food-based diet, and this may adversely affect body composition (BMD, lean mass/function, and fat mass/distribution) and cardiometabolic risk factors relative to slow weight loss. A systematic review comparing VLEDs and low energy diets (LEDs), which may respectively be defined as providing <3400 kJ (<800 kcal) per day as mentioned above [19], and between 3400–5000 kJ (800–1200 kcal) per day [32,33], showed that larger energy deficits induce greater losses of fat free mass in adults with overweight and obesity [34]. However, the studies included in that systematic review differed in total weight loss, duration of the intervention, and did not include studies where participants were randomly assigned to a VLED or LED. More recent studies have similarly shown significantly greater losses of lean body mass after fast weight loss (of at least 5% of initial body weight in 5 weeks) compared to after slow weight loss (of at least 5% of initial body weight in 15 weeks) in postmenopausal women [35] and in men and women [36] with overweight or obesity. Short-term outcomes of faster versus slower weight loss have also been reported in elite athletes, where fast weight loss of ~1.4% of body weight per week over 5.3 ± 0.9 weeks was associated with greater loss of lean mass and inferior performance on upper body strength tests compared to slower weight loss of ~0.7% of body weight per week over 8.5 ± 2.2 weeks [37]. However, responses to fast or slow weight loss could conceivably be different between elite athletes and people with overweight and obesity. To our knowledge, no study has directly compared the long-term effects of fast weight loss (achieved via VLED in a total meal replacement diet involving severe energy restriction) in comparison to slow weight loss (achieved via a conventional food-based diet involving moderate energy restriction) on body composition in people with obesity.

Therefore, the aim of the current work is to make a head-to-head comparison of the long-term (3-year) effects of fast versus slow weight loss, notably with respect to body composition, but also on eating behavior. This is addressed in the randomized controlled TEMPO Diet Trial (**T**ype of **E**nergy **M**anipulation for **P**romoting optimum metabolic health and body composition in **O**besity), the protocol for which is described here.

## 2. Materials and Methods

### 2.1. Ethics and Participants

The TEMPO Diet Trial is a randomized controlled trial designed to assess the long-term effects of fast versus slow weight loss on body composition and cardiometabolic health in postmenopausal women aged 45–65 years and with a BMI of 30–40 kg/m^2^. The standardized inclusion and exclusion criteria for the TEMPO Diet Trial are listed in Table 1. Our target sample size of 100 participants was determined based on power calculations with body composition as the primary outcome, and allowing for up to 20% attrition as seen in previously-published weight loss interventions [38,39,40]. Multiple strategies were used to fully recruit participants into the trial (*n* = 101) from March 2013 to July 2016. Recruitment strategies included word of mouth, free publicity on radio and TV, printed advertorials, internet-based advertisements, clinical trial databases and intranets, flyer distribution at community events, hospitals, the local tertiary education campus, local health service centers and residential mailboxes, as well as referrals from healthcare professionals. Full details of the recruitment process and recruitment strategies for the trial have been published previously [41].

Ethical approval was obtained from the Sydney Local Health District, Royal Prince Alfred Hospital Human Research Ethics Committee. The trial was prospectively registered with the Australian New Zealand Clinical Trials Registry (ANZCTR Reference Number 12612000651886). It is being conducted at the Charles Perkins Centre Royal Prince Alfred Clinic on the University of Sydney campus in Camperdown, NSW, Australia, with Magnetic Resonance Imaging (MRI) scans being performed at I-Med Radiology, Camperdown, NSW, Australia.

### 2.2. Interventions

Eligible participants were randomized to either a “FAST” or “SLOW” weight loss intervention. The FAST intervention involved a total meal replacement diet (KicStart^TM ^meal replacement products [shakes and soups] from Prima Health Solutions Pty Ltd., Brookvale, NSW, Australia) involving severe energy restriction (target range for energy restriction was 65–75% relative to estimated energy expenditure) for 16 weeks, or until a BMI of no lower than 20 kg/m^2^ was reached, whichever came first, followed by slow weight loss (as for the SLOW intervention) for the remaining time up until 52 weeks (FAST intervention). The SLOW intervention involved a food-based, moderately energy-restricted diet (target range for energy restriction was 25–35% relative to estimated energy expenditure) for a total of 52 weeks. As such, we aimed for a minimum difference in energy restriction between women in the FAST versus SLOW interventions of 30% of estimated energy expenditure. The SLOW intervention was based on the Australian Guide to Healthy Eating (AGHE) [46], which provides recommendations as to the average number of standard serves of the five core food groups (vegetables, fruits, grains and cereals, meat and meat alternatives, reduced fat dairy) an individual should consume in order to meet their nutritional requirements, based on age and sex. In order to simplify adherence to the SLOW intervention in the TEMPO Diet Trial we defined six food groups. The meat and meat alternative and reduced fat dairy core food groups were collapsed into a ‘proteins’ group, and starchy vegetables were incorporated into the grains and cereals group to form a ‘carbohydrates’ group while also having a vegetables, fruits, fats, and discretionary foods group. For the FAST intervention, prescribed daily carbohydrate intake was less than 100 g. A protein intake of 1.0 g per kg of actual body weight per day was prescribed for both interventions. As it is not possible to achieve a protein intake of 1.0 g per kg using meal replacement products alone, without increasing energy and carbohydrate intake above target levels for the FAST intervention, the total meal replacement diet (KicStart^TM^) was supplemented with a whey protein isolate (Beneprotein^®^, Nestlé HealthCare Nutrition Inc, Florham Park, NJ, USA). The development process and rationale behind the dietary interventions for the TEMPO Diet Trial, as well as full details of the dietary interventions, have been published previously [47].

### 2.3. Study Protocol

Two weeks prior to commencement of the dietary interventions (−2 weeks), eligible prospective participants attended our clinical research facility for interim measurement of weight, and for administration of several questionnaires and an interview related to the outcomes shown at −2 weeks in Table 2. They were instructed to maintain a steady weight (±1 kg) until 0 weeks, and two more face-to-face appointments were scheduled (one at −1 weeks, and another at 0 weeks). During this period, participants were requested to complete a food, activity and sleep diary for 7 days, to wear a pedometer and activity monitor for 7 days, to weigh themselves every morning at home before breakfast and record the results, to purchase an athletic bra and leggings suitable for use in the trial during the anthropometry and body composition measurements, and to bring all data and materials to the face-to-face appointments at −1 and 0 weeks. This procedure was implemented to not only help reduce variability in outcome measures at 0 weeks by facilitating weight stability, but also to help ensure that participants who continued to −1 weeks and beyond were likely to comply with trial requirements. It should be noted that all prospective participants successfully completed all requested tasks by −1 weeks and proceeded to randomization at that time point as described below, marking their official enrolment into the trial. At −1 weeks participants met with the trial dietitian (A.A.G. or C.H.) for a detailed weight history and counselling on following the diet to which they had been randomized, and certain outcome measures were undertaken or commenced at this time point.

Table 2 lists the schedule of all outcome measures for the trial. In brief, there are 8–9 major time points over the 3-year trial (0, 0.25, 1, 4, (and 4.25 for those in the FAST intervention), 6, 12, 24, and 36 months relative to commencement of the dietary interventions), with measurements undertaken during 1–3 face-to-face appointments at our clinical research facility, ideally scheduled at ±2 weeks of these major time points as shown in Table 2. We undertook measurements over 1–3 face-to-face appointments at each major time point because the measurements took up to 13 h to complete (which would have been too burdensome for participants if conducted in a single appointment of greater than 7 h in duration), and because some measurements (i.e., those for which participants were given a food, activity and sleep diary to complete for 7 days, and were requested to wear a pedometer and an activity monitor for 7 days) required meeting with participants a week before in order to provide materials and instructions for data collection over the ensuing week. Participants were given medical certificates to enable them to take leave of absence from work for trial appointments (if required), and—while in our clinical research facility—they were given a meal every 3–4 h, or a coffee voucher to use in the adjacent café during breaks from testing. The measurements at 0 and 0.25 months (0 and 1 weeks), as well as those at 4 and 4.25 months (16 and 17 weeks), were always done exactly 1 week apart. The measurements at 1 month were done within ±2 days from the exact date. The measurements at 4 and 6 months were done within ±1 week from the exact date. The measurements at 12 months were done within ±2 weeks from the exact date, while the measurements at 24 and 36 months were done within ±4 weeks from the exact date.

In addition to attending our clinical research facility for data collection, participants were required to attend 21–22 individual dietary appointments with the trial dietitian (A.A.G. or C.H.). The initial individual dietary appointment at week 0 was scheduled for approximately 90 min, which is also when participants in the FAST intervention received their first meal replacement products (shakes and soups) and protein supplementation as required [47]. Subsequent individual dietary appointments (for review) were scheduled for 30 min approximately every 2 weeks for the first 26 weeks of the intervention (i.e., at 1, 2, 4, 6, 8, 10, 12, 15, 16, 18, 20, 25, and 26 weeks relative to commencement of the dietary interventions, plus an extra appointment at 17 weeks for participants in the FAST intervention during their transition from the FAST to the SLOW intervention), and then approximately every month until 52 weeks (i.e., at 29, 33, 37, 41, 45, 51, and 52 weeks). To increase compliance with individual dietary appointments, participants were able to complete appointments that did not require face-to-face contact (i.e., to collect a food, activity, and sleep diary or to collect meal replacement products and protein) via telephone. After 52 weeks, participants were given the option of attending monthly group support meetings of 60–90 min in duration each, facilitated on a rotating basis by different members of the research team (R.V.S., A.A.G., C.H., F.Q.d.L. and A.S.), sometimes in association with a guest facilitator.

### 2.4. Randomization

At −1 weeks, participants were randomized (and enrolled) into the trial using stratified permuted block randomization [48]. Specifically, they were stratified by BMI (30–34.9 kg/m^2^; 35–40 kg/m^2^) and age (45–54.9 years; 55–65 years). Individuals in each of the 4 stratified groups were then randomized in blocks of 2 and with a 1:1 ratio into the FAST or SLOW intervention. To avoid bias, randomization was undertaken by an investigator (A.S.) who had not had contact with participants before randomization and was not involved in implementation of the 52-week dietary interventions. Researchers undertaking screening (R.V.S., A.A.G., C.H.) and dietary interventions (A.A.G., C.H.) were not aware of the method used for randomization and were not able to predict which intervention a particular participant would be randomized to.

### 2.5. Preparing Participants Before Outcome Measurements

One week prior to each face-to-face appointment at our clinical research facility, detailed instructions were e-mailed to each participant regarding what they needed to do prior to the day of the appointment as well as on the appointment day itself. At all appointments that involved measurement of weight and blood or urine sampling (i.e., at 0, 1, 4, 16 [and 17 for those in the FAST intervention], 26, 52, 104, and 156 weeks as shown in Table 2), participants were asked to attend our clinical research facility after an overnight fast (eight hours or more). They were instructed to drink plenty of fluid the day before so that they would be adequately hydrated on the day. In preparation for urine sample collection, participants were asked to drink a glass of water (250 mL) and to void their bladder once at home before attending our clinical research facility. They were also instructed to minimize physical activity on the morning of the appointment day, to minimize possible confounding of resting energy expenditure measurement.

### 2.6. Outcome Measures

All anthropometry and body composition data were collected with participants lightly-clothed (i.e., in the close-fitting sports bra and leggings that they purchased for use in the trial), without shoes, and with all metal jewelry, accessories, and electronic devices removed.

#### 2.6.1. Body Composition

##### Height

Height was measured to the nearest 0.1 cm using a stadiometer (Harpenden Stadiometer, Holtain Ltd., Crymych, UK). Two measurements were taken, and if the difference between the measurements was >0.5 cm, a third measurement was taken. The average of the two measurements (or the average of the two closest measurements if a third measurement was taken) was recorded as the result.

##### Weight (and BMI)

Body weight was measured to the nearest 0.1 kg with a calibrated scale (Tanita BWB-800 digital scale, Wedderburn Pty Ltd., Sydney, Australia). Two measurements were taken, and if the difference between the measurements was >0.5 kg, a third measurement was taken. The average of the two measurements (or the average of the two closest measurements if a third measurement was taken) was recorded as the result. BMI (kg/m^2^) was calculated by dividing weight (in kg) by height (in m) squared.

##### Bone

Bone Mineral Density (BMD) and Bone Mineral Content (BMC)

A dual-energy X-ray absorptiometry (DXA) machine (Discovery W model, Hologic Inc, Bedford, MA, USA) was used to measure BMD (g/cm^2^) and BMC (g) of the total left hip and anterior posterior lumbar spine (L1-L4). Scans were conducted in accordance with the recommendations outlined in the manufacturer’s manual and were assisted by use of the Hologic hip positioning fixture and a large square cushion for the hip and spine scans, respectively. Scans were analyzed using the integrated Hologic APEX Software (version 4, Hologic Inc, Bedford, MA, USA). Regions of interest were manually inspected, with adjustments where necessary. The DXA machine was serviced annually, subject to regular quality assurance testing, and calibrated on each day of use with a Hologic phantom spine.

Markers of Bone Turnover

Markers of Bone Formation

For the subsequent analysis of serum N-terminal propeptide of type I procollagen (P1NP) and osteocalcin, an intravenous cannula was inserted into a forearm vein and venous blood samples were collected into serum tubes from Becton Dickinson (BD, Australia New Zealand, North Ryde, NSW, Australia) and allowed to clot at room temperature. Serum was obtained by centrifugation of blood samples at 3200 rpm (~1600 × *g*) for 10 min at 4 °C (Eppendorf Centrifuge 5702, Eppendorf AG, Hamburg, Germany). Serum aliquots were pipetted into CryoPure^®^ tubes (Sarstedt Australia, Technology Park, SA, Australia) and immediately stored at −80 °C until analysis.

Markers of Bone Resorption

For the subsequent analysis of C-terminal telopeptide of type I collagen (CTX), venous blood samples were collected into serum tubes and serum was prepared and stored as described above until subsequent analysis. In addition, a fasting urine sample was collected from participants and pipetted into a CryoPure^®^ tube and immediately stored at −80 °C until subsequent analysis of N-terminal telopeptide of type I collagen (NTX).

##### Lean Tissues

Fat Free Mass (FFM, DXA)

A whole-body DXA scan was carried out to quantify FFM (kg) and fat mass (FM) (kg and % of body weight), using the DXA machine as described above. During the scan, participants lay supine on the scanning table with their arms at their sides. Scans were conducted in accordance with the procedure outlined in the manufacturer’s manual and were analyzed using the integrated Hologic APEX Software, which was used to define regions of the body (i.e., head, arms, trunk, and legs).

Fat Free Mass (4-Compartment Model)

The 4-compartment model was used because it is the most accurate way to assess body composition under conditions (such as weight loss) where body composition is changing [49,50,51]. This gold-standard method can be explained by considering the body to be composed of a variety of “compartments” which include bone, fat, water, and a “residual” compartment that is largely made up of protein (mostly muscle) but which also includes non-bone mineral and glycogen. When estimating body composition, the accuracy of the measurement depends on how many compartments can be measured directly, with each direct measurement reducing the number of assumptions that need to be made. For example, methods that utilize a 2-compartment model, such as bioelectrical impedance, generally separate the body into a fat compartment and a fat free compartment. Because this method directly measures only the fat compartment, not the fat free compartment, a number of assumptions are made for the estimation of FFM, especially regarding its density, which is highly variable depending on the relative proportions of muscle mass, BMC, and total body water (TBW). All of these variables change from one person to the next, and also within the same person under different conditions (e.g., before and after weight loss). Methods that utilize a 3-compartment model, such as DXA, air displacement plethysmography and underwater weighing, are more accurate than methods that draw on a 2-compartment model, but none of these methods control for inter-individual variability (and intra-individual variability over time) in both BMC *and* TBW.

With the 4-compartment model, direct measurements of BMC using DXA, body volume using air displacement plethysmography or underwater weighing, and TBW using the deuterium dilution technique, reduces the number of assumptions that need to be made when calculating body composition, because the masses of most of the components of the fat free compartment are measured rather than assumed. FFM (and FM) using the 4-compartment model can be calculated from the equations below [52]:

FFM (kg) = body weight (kg) − FM (kg)(1)

FM (kg) = [2.748 × body volume (L)] − [0.699 × TBW (kg)] + [1.129 × BMC (kg)] − 2.051 × body weight (kg)](2)

Body Volume (BodPod)

As described above, the 4-compartment model requires body volume to calculate FFM (and FM), and this was measured via air displacement plethysmography using a BOD POD^®^ (COSMED USA Inc, Concord, CA, USA) following the manufacturer’s recommended procedures. In addition to the attire described above for anthropometry and body composition assessment, each participant also wore a Lycra swim cap during body volume measurement. After initial calibration of the BodPod^®^, participants were weighed on an electronic scale. The resulting body weight, as well as sex, age, and height, were entered into the BodPod^®^ software system (BOD POD version 5.4.3) to estimate (predict) thoracic lung volume. Participants were given a brief description of the procedure before entering the BodPod^®^ chamber for the first of two sequential body volume measurements. If the difference between these two measurements was >0.150 L, a third measurement was taken. The average of the two measurements (or the average of the two closest measurements if a third measurement was taken) was recorded as the result for body volume.

Total Body Water (TBW, Deuterium Dilution)

Stock deuterium oxide (99.9% atom % D, Sigma-Aldrich Co, St Louis, MO, USA) was diluted to a 10% dosing solution (*v*/*v*) by adding 100 mL of the stock deuterium oxide to 900 mL of tap water. The 10% deuterium oxide dosing solution was then autoclaved and stored at −4 °C. When arriving at the clinical research facility on testing days after an overnight-fast, participants were instructed to first void the bladder (and in doing so also collect a urine sample, the ‘pre-dose urine sample’). Participants were then given a dose of 0.5 g per kg of body weight of the 10% deuterium oxide dosing solution in a cup with a straw. The weight of the cup + straw, and the weight of the cup + straw + calculated dose of 10% deuterium oxide, were recorded. Participants were asked to ingest as much of the liquid as they could using the straw provided, and then the weight of the cup + straw was recorded again to account for any leftover 10% deuterium oxide dose remaining in the cup. After a six-hour equilibrium period, during which time participants were given a total of 650 mL of tap water to drink, participants were asked to collect another urine sample (the ‘post-dose urine sample’). All urine samples were labelled and stored at −80 °C before processing for analysis of deuterium content. To this end, 5 mL of each urine sample was passed through 200 mg of charcoal to remove organic matter, and 1 mL of the filtrate was pipetted into clear 2 mL screw-top vials sealed with caps containing a hole with a polytetrafluoroethylene/silicone septum (product numbers SV08CW and AC08WK, respectively, Pacific Laboratory Products, Victoria, Australia). Cavity ring-down spectroscopy was used to determine the abundance of deuterium in the processed urine samples, as well as in the tap water and a sample of the 10% deuterium oxide dosing solution (which was diluted 1 in 100 with tap water to make a 0.1% dilution of the original stock deuterium oxide), using a Picarro L2130-i Isotopic Water Analyzer (Picarro Inc, Santa Clara, CA, USA). TBW was calculated using the following equation [53]:

TBW = [(W × A)/a] × [(δdose − δtap)/(δpost − δpre)] × [1/(1000 × 1.041)](3)

W is weight of water used to further dilute the 10% deuterium oxide dosing solution to 0.1% (g)
A is weight of the 10% deuterium oxide dosing solution consumed by the participant (g)
a is weight of the 10% deuterium oxide dosing solution that was further diluted to 0.1% (g)
δdose is abundance of deuterium in the 0.1% deuterium oxide solution (ppm)
δtap is abundance of deuterium in the tap water used to make the dilutions (ppm)
δpost is abundance of deuterium in the processed post-dose urine sample (ppm)
δpre is abundance of deuterium in the processed pre-dose urine sample (ppm)

Thigh Muscle Cross Sectional Area (MRI)

Thigh muscle cross sectional area was determined using MRI with participants lying in a supine position and having removed any metal accessories. Axial images were acquired with a 3.0T MRI scanner (the Discovery MR750 3.0T model from GE Healthcare, Milwaukee, WI, USA), between the base of the femoral head and mid-patella (repetition time = 700 ms, echo time = 10 ms, flip angle = 111°) with a slice thickness of 1 cm and an inter-slice gap (i.e., the distance between the surfaces of adjacent slices) of 1 cm. For subsequent analysis the median slice between these two points was segmented manually using specialist analysis software (SliceOMatic Version 5.0 rev-6b, Tomovision Inc, Montreal, QC, Canada). When there was an even number of slices between these two points, the inferior of the two slices was selected for analysis. Total skeletal muscle area (and volume) of the thigh muscles (rectus femoris, vastus lateralis, vastus intermedius, vastus medialis, satorius, biceps femoris short head, biceps femoris long head, semitendinosis, semimembranosis, gracilis, and the adductor group) was defined using the region-growing mode of the software, with thresholds adjusted manually as required. Analysis was undertaken by a trained experimenter blinded to treatment allocation.

Muscle Strength (Hand Dynamometry)

Handgrip strength was assessed using a commercially-available hydraulic hand dynamometer (Jamar^®^, Model 5030J1, Patterson Medical, Bolingbrook, IL, USA) in accordance with a standardized protocol [54]. In brief, participants were seated upright in an armed chair, with both feet flat on the ground, their forearm resting on the chair arm with an elbow angle of 90° and their wrist positioned over the edge of the chair arm. Participants were instructed to grip the hand dynamometer by the handle and to squeeze as hard as they could. The investigator provided verbal encouragement, and the peak value was recorded to the nearest 1 kg. Three measurements were alternatively recorded for both the left and the right hand, and the maximum reading of the three measurements was used as the result for each hand. A one-minute interval was timed between subsequent measures of the same hand to avoid fatigue. Participants were asked to identify their dominant hand, which was recorded, and handgrip strength was recorded as dominant and non-dominant handgrip strength.

##### Fat Mass and Distribution

Waist and Hip Circumference (and Ratio of Waist to Hip Circumference)

Waist and hip circumferences were measured to the nearest 0.1 cm using a narrow, flexible, and inelastic steel tape (Lufkin W606PM, Apex Tool Group, NC, USA). Participants were asked to breathe normally and to stand with their weight evenly distributed and their arms crossed across their chest during measurements. Waist circumference was measured directly on the skin, at each of the three most commonly used sites worldwide [55]; at the mid-axillary line (i.e., at the halfway point between the bony landmarks of the lowest rib and the top of the iliac crest); at the narrowest part of the torso, and at the level of the umbilicus. Hip circumference measurement was taken at the point of greatest protuberance of the participant’s buttocks when viewed from the side. At each site, two measurements were taken, and if the difference between the measurements was >1 cm, a third measurement was taken. The average of each of the two measurements at each site (or the average of the two closest measurements if a third measurement was taken) was recorded as the result.

Fat Mass (DXA) and Fat Mass (4-Compartment Model)

As described above with FFM (DXA) and FFM (4-compartment mode).

Abdominal Fat (MRI)

Abdominal fat volumes, namely visceral adipose tissue (VAT) and subcutaneous adipose tissue (SAT) volumes, were quantified using MRI as described above for the section entitled *Thigh Muscle Cross Sectional Area*. Axial T1-weighted fast field echo images were acquired from diaphragm to pelvis (repetition time = 3.8 ms, echo time = 2.1 ms, flip angle = 12°), with a slice thickness of 10 mm and an inter-slice gap of 10 mm. Images were acquired during suspended end-expiration, with a breath-hold duration of approximately 15–18 s per acquisition. Following the scan, all image slices from the base of the lungs to the pelvic floor were segmented manually. VAT was quantified using the Region Growing mode of the analysis software (SliceOMatic Version 5.0 rev-6b, Tomovision Inc, Montreal, QC, Canada), and SAT was quantified using the Mathematical Morphology mode of the software, with thresholds adjusted manually as required. The software was used to automatically calculate the surface area of VAT and SAT on each slice by multiplying the number of pixels tagged by the surface area of one pixel. The inter-slice volume (i.e., the volume of the inter-slice gap) was extrapolated using a cone formula that considered the surface area of the superior and inferior surfaces of the inter-slice gap, as well as the thickness of the inter-slice gap. The total volume of each of the inter-slice gaps was then summed to the total volume of each of the slices (surface area × slice thickness) to calculate the total abdominal volumes of VAT and SAT.

Intrahepatic Lipid (^1^H-MRS)

Intrahepatic lipid was measured by proton magnetic resonance spectroscopy (^1^H-MRS) using a 3.0T MRI scanner (Discovery MR750 3.0T from GE Healthcare, Milwaukee, WI, USA) and analyzed by an assessor blinded to intervention group. Intrahepatic lipid was analyzed by image-guided, localized ^1^H-MRS with a voxel of 30 mm × 30 mm × 20 mm using the whole-body (Q body) coil and a 32-channel torso array coil (NeoCoil), with volumes of interest centered in the right lobe of the liver. Participants lay supine, with spectra acquired during respiratory gating. Spectra were acquired using the PRESS (point resolved spectroscopy) technique (TR = 3000–6000 ms dependent on participant respiration rate, TE = 35 ms, 32 measurements, 4092 sample points). Placement of the voxel in the liver was kept as consistent as possible between measures within each participant, by noting (via image capture) the location of the voxel at the first measurement time point (i.e., at −1 weeks), and replicating this placement as closely as possible in subsequent measurement time points in the same participant. Variation was further minimized by the use of a large voxel, and we have previously shown that the coefficient of variation for this technique is approximately 7% [56]. Fully automated high-order shimming was performed on the volume of interest to ensure maximum field homogeneity. The in vivo water signal was used as the internal standard. Spectral data were post-processed by magnetic resonance user interface software (jMRUI version 5.2, EU Project). Hepatic water signal amplitudes were measured from the acquired non-water suppressed spectra using HLSSVD (Hankel Lanczos Squared Singular Values Decomposition). For hepatic lipid concentration and composition, a five-resonance model was employed [56]. Resonances were fitted for water (4.7 ppm) and fatty acid functional groups: diallylic (2.8–3.1 ppm), other lipids (2.0–2.4 ppm), methylene (1.2–1.4 ppm), and methyl (0.8–1.0 ppm). The signal amplitude was obtained in absolute units for each resonance using AMARES fitting (Advanced Method for Accurate, Robust, and Efficient Spectral), a nonlinear least squares quantitation algorithm, as previously described [57]. Peak amplitudes were determined using prior knowledge that we have detailed previously [56]. The resonances were fitted assuming a Gaussian line shape for all lipid resonances. The zero-order and first-order phase corrections were manually estimated.

Thigh Fat (MRI)

The MRI scans of the thigh that were described under the section entitled *Thigh Muscle Cross Sectional Area* (above) were used to quantify three types of thigh fat using specialist analysis software (SliceOMatic Version 5.0 rev-6b, Tomovision Inc, Montreal, QC, Canada): SAT of the thigh, subfascial adipose tissue, and intermuscular adipose tissue. SAT of the thigh was identified using the Mathematical Morphology mode of the software as described above for SAT under the heading of *Abdominal Fat*, while the two other adipose tissue compartments—subfascial adipose tissue and intermuscular adipose tissue—were defined using the Region Growing mode of the analysis software, with thresholds adjusted manually as required.

#### 2.6.2. Cardiometabolic Health (in Addition to Waist Circumference)

##### Blood Pressure

Blood pressure was measured using an automated device from Welch Allyn Medical Products (Skaneateles Falls, NY, USA). Participants rested in a sitting position for two minutes before two measurements were recorded for both the left and right arms. The average of the two recordings was used as the result for each arm.

##### Circulating Markers of Cardiometabolic Health

Venous blood was collected and serum prepared and stored as described above for the section entitled *Markers of Bone Turnover*, for subsequent analysis of glucose, insulin, triglycerides, and cholesterols. Whole venous blood was collected directly into a CryoPure^®^ tube and frozen at −80 °C until subsequent analysis of glycosylated haemoglobin.

##### Vascular Function and Structure

Flow Mediated Dilatation (FMD)

Ultrasound assessment of brachial artery FMD was performed on the right arm in a slightly darkened, temperature-controlled room using an EPIQ 7 Ultrasound System with an L12-3 linear array transducer (Philips, Bothell, WA, USA). Simultaneous electrocardiogram (ECG) recordings were made throughout. The brachial artery was scanned in longitudinal sections 2–10 cm above the antecubital fossa. Ultrasound images were recorded for 30 s at rest, and arterial flow velocity was measured with a pulsed Doppler signal. Increased blood flow was induced by inflation of a blood pressure cuff placed around the forearm, to a suprasystolic pressure of at least 50 mmHg above resting systolic blood pressure. This occlusion was maintained for 5 min, and then the cuff was deflated. Within the first 15 s after the pressure from the cuff was released, a pulsed Doppler velocity signal was acquired to assess the post-occlusive hyperemia. B-mode ultrasound was recorded from 30 s to 2 min post-occlusion, and saved in the DICOM format (Digital Imaging and Communications in Medicine). Brachial artery diameter was subsequently measured offline by a reader blinded to participant characteristics and time point, using semi-automated software (Brachial Analyzer for Research, Medical Imaging Applications LLC, Coralville, IA, USA). Dilatation at 50–70 s after cuff release (FMD60), and the maximum percentage increase in brachial artery diameter in response to hyperemia (%FMD), was recorded.

Carotid Intima Media Thickness (cIMT)

cIMT is a validated non-invasive marker of subclinical atherosclerosis in adults [58]. The procedure was conducted in the same slightly darkened room as FMD, immediately before the FMD measures, with the participant resting in a supine position with their neck slightly hyperextended and rotated 45°. B-mode ultrasound images were acquired using the EPIQ 7 Ultrasound System with a L12-3 linear array transducer as described above. Both the left and the right common carotid arteries were visualized just proximal to the bulb, in the longitudinal plane and at three distinct angles each (a total of six angles; 90°, 120°, 150°, 210°, 240°, and 270°), identified using a Meijer’s Arc. Two 10-s loops were acquired for each angle (12 loops in total per time point for each participant). B-mode images were saved in the DICOM format and imported into a semi-automated program for analysis of cIMT (Carotid Analyzer for Research, Medical Imaging Applications LLC, Coralville, IA, USA). The best quality loop from each angle was analyzed, with the region of interest placed 5–10 mm proximal to the start of the carotid bulb. Mean cIMT, maximum cIMT, and diameter were measured. The mean of the mean cIMT measures from all 6 angles was calculated, as was the mean of the maximum cIMT from all 6 angles.

#### 2.6.3. Eating Disorder Behaviors/Psychology

##### Eating Disorders Examination (EDE)

The EDE-v16 is an investigator-led interview that is considered the gold standard instrument for the assessment of eating disorders [59]. It is used to assess eating disorder behaviors (i.e., binge eating, purging, very strict dieting) and eating disorder psychopathology, namely restraint, eating concern, weight, and shape overvaluation. It has robust psychometric qualities. A global score can be derived of dietary restraint, eating and weight/shape cognitions.

##### Eating Disorders Examination Questionnaire (EDE-Q)

The EDE-Q*-*v6 modified, is the self-reported questionnaire based on the gold standard EDE for the assessment of eating disorder behaviors and psychopathology [60,61]. In order to exemplify the concepts in the EDE-Q (e.g., objective binge eating) to participants, an initial explanatory text was presented before participants answered the EDE-Q. This initial explanatory text was published in another study [62]. Additionally, we added a question regarding subjective binge eating, as per a previous publication [63]: “Have you had other episodes of eating in which you have had a sense of having lost control and eaten more than you would like, but *have not eaten a very large amount* of food given the situation? Over the past 28 days how many *days* approximately would this have happened?” We also replaced the words “16 ounce” with “450 g” due to this questionnaire being administered in Australia where the metric (rather than the imperial) system is used. Questions regarding weight, height, and menstrual periods were removed because weight and height are available from laboratory measurements, and because all participants in the TEMPO Diet Trial are postmenopausal women.

##### Loss of Control over Eating Scale (LOCES)

The experience of loss of control over eating constitutes a clinically significant feature of eating disorders. However, this feature is assessed only in a dichotomous “yes or no” manner in the EDE and EDE-Q, and this may lead to imprecise assessments. Therefore, the LOCES was used in the current trial to complement assessments from the EDE and EDE-Q. The LOCES is a scale used to assess this essential feature of eating disorders, namely the experience of loss of control over eating [64] in both clinical and non-clinical samples [65].

##### Three Factor Eating Questionnaire (TFEQ)

The TFEQ is a self-report 51-item questionnaire used to assess three aspects of eating behavior, namely “cognitive restraint of eating”, “disinhibition” and “hunger” [66,67]. Two items (item 1 and 4) on the questionnaire were modified: (1) “When I smell a sizzling steak or a juicy piece of meat, I find it very difficult to keep from eating, even if I have just finished a meal” was changed to “When I smell a sizzling steak *or some delicious savory food*, I find it very difficult to keep from eating, even if I have just finished a meal”, to make the question appropriate for vegetarians; (4) “When I have eaten my quota of calories, I am usually good about not eating anymore” was changed to “When I have eaten my quota of calories */ kilojoules*, I am usually good about not eating anymore” [68], because an increasing number of individuals in Australia count kilojoules (metric) instead of calories (imperial).

##### Dutch Eating Behaviour Questionnaire (DEBQ)

The DEBQ is a self-report 31-item questionnaire that contains three scales that assess three different types of eating behavior, namely “restrained eating”, “emotional eating” and “external eating” [69,70].

##### Mini International Neuropsychiatric Interview (MINI)

All Questions

The MINI (version 7.0.0) [71] is a structured interview used to assess the occurrence of psychiatric disorders in accordance with the criteria of the Diagnostic and Statistical Manual of Mental Disorders fifth edition (DSM-5) [64]. In the current trial, the complete MINI (i.e., all questions) was only administered once, at −2 weeks.

Lifetime History of Eating Disorder Questions

Questions from the MINI (version 7.0.0) that assess binge eating disorder, bulimia nervosa and anorexia nervosa were adapted to assess the lifetime occurrence of these eating disorders (that is, whether a participant had any of these disorders at any time in the past), using the DSM-5 criteria.

Eating Disorder Questions Only

At all other time points (besides the −2-week time point) where the MINI was administered (i.e., at 29, 51, 103, and 154 weeks as shown in Table 2), only the sections of the MINI (version 7.0.0) that assess binge eating disorder and bulimia nervosa was used.

##### General Mental Health

Rosenberg Self-Esteem Scale

This instrument assesses self-worth by measuring both negative and positive feelings regarding the self. This scale contains 10 items that are answered in a 4-point Likert scale that ranges from “Strongly agree” to “Strongly disagree” [72].

36-Item Short Form Survey (SF-36)

The SF-36 is a self-report survey used to assess health status regarding pain, mental health, vitality, general health perception, and limitations in usual activities because of physical or emotional problems [73].

Depression, Anxiety and Stress Scale (DASS-21)

The DASS-21 is a self-report scale with 21 items used to assess symptoms of depression, anxiety and psychological stress in both clinical and non-clinical samples [74,75,76].

##### Mindfulness and Personality

Langer Mindfulness Scale (LMS)

The LMS is a 14-item questionnaire that assesses three sub facets of mindfulness: (1) novelty seeking (i.e., having an open and curious orientation to the environment), (2) novelty producing (i.e., an individual’s tendency to create new categories rather than relying on those previously formed) and (3) engagement (i.e., the ability to attend to changes in the environment) [77]. Participants are asked to answer the items on a 7-point Likert scale ranging from “Disagree” to “Agree”. The LMS has adequate internal consistency and temporal stability, and correlates appropriately with other instruments assessing theoretically-related constructs [78].

Five Facet Mindfulness Questionnaire

This 39-item self-report questionnaire was used to assess five different facets of mindfulness, namely observing, describing, acting with awareness, non-judging of inner experience, and non-reactivity to inner experience [79].

Big Five Inventory

This is used to measure five different dimensions of personality and each of these five different dimensions of personality is subdivided into six personality facets [80,81,82].

#### 2.6.4. Adaptive Responses to Energy Restriction

##### Energy Intake, and Factors Influencing it

Food Diary

Participants were provided with detailed written and verbal instructions prior to completing 7-day estimated food diaries which were reviewed for completeness by a dietitian [83]. These food diaries were used for data collection, as well as to aid in administration of the weight loss interventions. The food diary that was collected from −1 to 0 weeks was used to tailor dietary counselling to each individual participant. At all other time points, the food diary was used to monitor adherence (i.e., servings of food groups, number of meal replacement products consumed per day, etc.), and to provide individualized feedback to participants about their progress. Food diaries were coded and analyzed for nutrient composition and food group serves by trained dietitians, using a standardized protocol and FoodWorks Professional version 8 (Xyris Software, Brisbane, QLD, Australia).

Drive to Eat

General Food Craving Questionnaire—State (G-FCQ-S)

The G-FCQ-S assesses state-dependent cravings for tasty foods in general. That is, it assesses whether general food cravings are experienced in response to specific, momentary situations or psychological and physiological states [84].

Eating Self-Efficacy Scale (ESES)

The ESES is a 25-item scale that rates the likelihood of having difficulty controlling overeating across two factors; one concerned with eating when experiencing negative affect; and the other with eating during socially acceptable circumstances [85,86]. Two items (item 1 and 10) on the questionnaire were modified: (1) “Overeating after work or school” was changed to “Overeating after work or school *(or after your day’s activities, if you do not go to work or school)*”, as the original version may not be applicable to participants that are not working or at school; (10) “Overeating with family members” was changed to “Overeating with family members *(or with other people, if you do not eat with family members)*”, as the original version may not be applicable to participants that do not (regularly) eat with family members.

Subjective Drive to Eat (Visual Analogue Scales)—Fasting and Postprandial

Subjective appetite perceptions were measured at −20, 0, 15, 30, 45, 60, 90, 120 and 180 min in relation to completion of a standardized breakfast using an Electronic Appetite Rating System (EARS) that has been validated against the pen-and-paper version [87]. The breakfast consisted of a wholemeal English muffin (Tip Top, Enfield, NSW, Australia), 2 eggs (~60 g each, cooked by scrambling in a non-stick pan), 15 g of olive oil spread (Coles Supermarkets Australia Pty Ltd., Hawthorn East, Victoria, Australia), which was used to spread on the muffin and to scramble the eggs, 200 mL orange juice (Just Juice, Docklands, Victoria, Australia) and 300 mL tap water. The meal amounted to ~700 g and ~1900 kJ (~450 kcal). Participants were asked to complete the meal within 10 min, and to rate perceptions of hunger, fullness, how much food they felt they could eat (prospective consumption), desire to eat, and satisfaction, using the EARS tool. Participants move the cursor along a horizontal 100 mm visual analogue scale, anchored at each end with the statements “Not at all” or “None at all” (0 mm) and “Extremely” or “A large amount” (100 mm), to a point on the horizontal line that reflects the intensity of their current state. Gastrointestinal and other symptoms, namely nausea, bloating, irritability, and alertness, were also assessed.

Appetite-Regulating Hormones—Postprandial and/or Fasting

The venous cannula that was inserted for collection of fasting blood samples (for determination of serum markers of bone turnover and cardiometabolic health) was used to collect blood for plasma preparation and determination of appetite-regulating hormones. Venous blood sample collections were commenced at −18, 32, 62, 122, and 182 min relative to completion of the above-mentioned standardized breakfast (i.e., immediately after measurement of subjective drive to eat, which took approximately 1 min). The blood was collected into ice-cold EDTA-treated tubes (BD, Australia New Zealand, North Ryde, NSW, Australia) containing inhibitors to prevent degradation of hormones. The inhibitors consisted of 1 mg Prefabloc^®^ (Roche Diagnostics, Castle Hill, NSW, Australia), 10 μL dipeptidyl peptidase-IV inhibitor (Merck Millipore, Burlington, MA, USA), and 10 μL protease inhibitor cocktail (Sigma-Aldrich, St Louis, Missouri, USA) per 1 mL whole blood. Immediately after collection, samples were plunged up to the neck into wet ice, and were centrifuged as described above in the section entitled *Markers of Bone Turnover*. Immediately after centrifugation, aliquots of plasma were stored at −80 °C until subsequent analysis of ghrelin and peptide YY (in fasting and postprandial plasma samples) and leptin (in fasting plasma samples only).

Ketones

β-Hydroxybutyrate (Whole Venous Blood)

Fasting venous blood from the cannula was used to measure concentrations of circulating ketones (β-hydroxybutyrate) using β-ketone test strips and a ketone monitor (FreeStyle Optium, Abbott Diabetes Care Ltd., Witney, Oxon, UK), as per the manufacturer’s protocol.

Acetoacetic Acid (Urine)

Urinary acetoacetic acid concentrations were estimated using over-the-counter reagent strips (Ketostix^®^, Bayer Vital GmbH, Leverkusen, Germany), which determine the presence of acetoacetic acid upon reaction with nitroprusside salt. The end of the strip was dipped into a fasting urine sample collected from the participant soon after they arrived at our clinical research facility. The colour of the reagent strip was then compared to the color chart provided with the product, at 15 s after contact with urine.

##### Physical Activity

Self-Efficacy to Regulate Exercise

This scale describes eighteen situations that can make it difficult to maintain an exercise routine, and assesses an individual’s confidence in their ability to perform their exercise routine regularly (three or more times a week) during these situations [85]. For each item, participants indicate their confidence to execute the behavior on a 100-point percentage scale comprised of 10-point increments, ranging from “Cannot do at all” (0%) to “highly certain can do” (100%).

Pedometer Step Count and Physical Activity Diary

Participants were given a calibrated pedometer (Omron Walking Stle HJ-203, Omron Healthcare Co Ltd., Kyoto, Japan) to be worn in a pocket or bra, and were advised to gradually increase daily step counts to a total of 8000–12,000 steps per day, including 30–60 min per day of moderate to vigorous physical activity. This recommendation was based on achieving 200–300 min per week of physical activity, as recommended in the 2009 American College of Sports Physicians physical activity guidelines for weight loss and prevention of weight regain in adults [88]. Participants were asked to record the number of steps displayed on their pedometer in a daily physical activity diary for 7 days. This was done in their food diary, which contained spaces for recording physical activity (and sleep). This diary was thus referred to as the food, activity, and sleep diary.

Accelerometry

Intensity and duration of daily physical activities, as well as sleep, were measured using an accelerometer (SenseWear Pro Armband^®^, BodyMedia Inc, Pittsburgh, PA, USA) [89]. Participants were asked to wear the accelerometer continuously for 7 days, on the back of the upper tricep muscle of the left arm, except when showering or for water-based activities (e.g., swimming) as the device is not water proof. Participants were asked to record the time and reasons for taking off the accelerometer in their food, activity, and sleep diary.

##### Sleep (in Addition to Accelerometry)

Sleep Diary

Participants were asked to record in their food, activity and sleep diary, for seven days, what time they woke up in the morning (which may be different from the time they actually got out of bed), what time they tried to go to sleep the previous night (which may be different from the time they got into bed), and approximately how long (in minutes) it took them to fall asleep the previous night.

Epsworth Sleepiness Scale (ESS)

The ESS consists of eight self-report items, each scored from 0–3, that measure a person’s habitual “likelihood of dozing or falling asleep” in common situations of daily living [90,91]. No specific time frame is specified. The ESS score represents the sum of individual items, and ranges from 0–24. Values >10 are considered to indicate significant sleepiness.

Pittsburgh Sleep Quality Index (PSQI)

The PSQI is a 19-item self-report questionnaire for evaluating subjective sleep quality over the previous month, combined into seven clinically-derived component scores, each weighted equally from 0–3 [92]. The seven component scores are added to obtain a global score ranging from 0–21, with higher scores indicating worse sleep quality.

##### Energy Expenditure

Resting Energy Expenditure (REE, Indirect Calorimetry)

REE was measured via indirect calorimetry using a ventilated hood system (TrueOne2400, ParvoMedics, Inc, Sandy, UT, USA) after an overnight fast. REE was measured with participants lying in a comfortable supine position, with their head on a pillow and a transparent ventilated hood placed over their head. Plastic sheeting attached to the hood was tucked around the participant to form a seal between the air inside and outside the hood. Participants lay in a quiet room with an ambient temperature of ~23–25 °C. The ventilated hood system was calibrated before each measurement using standardized gases and rates of airflow, according to the manufacturer’s manual. Volumes of oxygen inspired and carbon dioxide expired were recorded continuously for 45 min, and the first 10 min of data were disregarded, as during this time the participant is considered to be adapting to the hood. REE (kJ/day) was calculated using the modified Weir equation, as previously published [93].

Body Temperature

Body temperature was measured over seven days using the same accelerometer used to measure physical activity and sleep, as described above. Body temperature was measured as it provides an indication of energy expenditure in a variety of mammalian species, including humans [94].

##### Neuroendocrine Status

As the hypothalamo-pituitary-adrenal, -thyroid, -gonadotropic, and -somatotropic axes are influenced by weight loss and are known to influence body composition if sustained for several weeks [15], serial measurements of hormones within these axes will demonstrate whether there is any differential effect of the FAST versus SLOW intervention on the concentrations of hormones that could adversely affect body composition, as well as the duration of any such changes. A suite of hormones will be measured to allow a more accurate assessment of whether the activity of each axis has been differentially altered by either intervention. Venous blood samples were collected either into serum tubes (BD, Australia New Zealand, North Ryde, NSW, Australia) and allowed to clot at room temperature, or collected into ice-cold EDTA-treated tubes (BD) and immediately plunged up to the neck into wet ice. Serum and plasma were obtained by centrifugation of blood samples as described above in the section entitled *Markers of Bone Turnover*, and aliquots were pipetted into CryoPure^®^ tubes and immediately stored at −80 °C until analysis. Serum and plasma samples were used for analysis of cortisol and adrenocorticotropic hormone, respectively, as indices of activity of the hypothalamo-pituitary-adrenal axis. Serum samples were used for analysis of free triiodothyronine or 3,3′,5-triiodothyronine (T3), reverse T3, free thyroxine or 3,5,3′,5′-tetraiodothyronine (T4), and thyroid stimulating hormone (TSH), as indices of activity of the hypothalamo-pituitary-thyroid axis; estradiol and sex hormone binding globulin, as indices of activity of the hypothalamo-pituitary-gonadotropic axis; and insulin-like growth factor-1 and insulin-like growth factor binding proteins, as indices of activity of the hypothalamo-pituitary-somatotropic axis.

##### Miscellaneous

Energy Homeostasis Questionnaire

This was a custom-built, experimental questionnaire in which we aimed to determine whether an individual’s propensity to lose weight or keep it off, or to mount adaptive physiological responses to energy restriction or energy excess, could be predicted by self-report of traits such as hunger, energy levels or body temperature after periods of relatively reduced or increased energy intake.

#### 2.6.5. General

##### Preferred Intervention

We asked participants to select 1 of 3 statements that best reflected how they felt when they found out which of the two interventions they had been randomized to. The statements were “I was disappointed”, “I was pleased”, and “I would have been pleased to have been randomized to either of the two diets”, with the different options listed in random order for each participant. This was asked in order to ascertain whether being randomized to the preferred intervention had any effect on the amount of weight that a participant lost on that intervention.

##### Diet Side Effect Questionnaire

The aim of this custom-built questionnaire was to capture and quantitate any side effects from the FAST intervention.

### 2.7. Statistical Analyses

Data analysis will be conducted using intention to treat principles. Exploratory analyses will be performed using appropriate statistical methods, such as the generalized linear mixed-model, as deemed appropriate.

## 3. Discussion

The observed reticence of healthcare professionals to prescribe dietary obesity treatments that induce fast weight loss [28,30] suggests an underlying assumption that fast weight loss is less advisable than slow weight loss. Potential concerns about the safety of fast weight loss—notably for musculoskeletal integrity but also for eating disorder behaviors—may contribute to the low utilization of fast weight loss by healthcare professionals. Given the short- and longer-term effectiveness of VLEDs as a dietary obesity treatment [18,20,22,23,24,27], it is necessary to know whether any concerns about their safety are founded, as the escalating obesity epidemic mandates use of all safe and effective obesity treatment options.

## 4. Conclusions

To our knowledge, the TEMPO Diet Trial will be the first randomized controlled trial to compare the long-term effects of fast weight loss (achieved via VLED in a total meal replacement diet involving severe energy restriction) to slow weight loss (achieved via a conventional food-based diet involving moderate energy restriction) on gold-standard measures of musculoskeletal integrity and eating disorder behaviors. This work has the potential to advance the treatment of obesity by providing information about the safety or otherwise of dietary obesity treatments that induce fast versus slow weight loss.

## Figures and Tables

**Table 1:** Inclusion and exclusion criteria for the TEMPO Diet Trial.

**Inclusion Criteria**
Female, due to the estimated lifetime risk of osteoporotic fractures being 3-fold higher in women than in men (40% versus 13%) [42]
45–65 years of age
Postmenopausal for ≥5 years (calculated from date of last menses), to circumvent known effects of female sex hormone cycles and the menopausal transition on parameters under investigation
Body mass index (BMI) 30–40 kg/m^2^
Weight stable (±2 kg) for ≥past 6 months
English-speaking
Living in the Sydney metropolitan area (defined by the City of Sydney Statistical Division [43,44]) and able to attend all in-person appointments at the University of Sydney Camperdown campus
Sedentary (defined as <3 h of structured physical activity per week)
Consider themselves capable of completing activities required for the trial (e.g., keeping a food, activity, and sleep diary, wearing accelerometers for seven days at a time, etc.)
**Exclusion Criteria**
Not ambulatory, or having restrictions to physical movement that would impede completion of trial activities
Osteoporosis
Extreme anemia that could be exacerbated by very low energy/total meal replacement diet
Hyperthyroidism or hypothyroidism
Diabetes mellitus (defined by fasting blood glucose level ≥7.0 mmol/L and glycated hemoglobin (HbA_1c_) ≥6.5%) [45]
Cardiovascular disease
Gastrointestinal disease
Previous gastric or other surgery that may affect appetite
Any loose metal in the body (e.g., pacemaker or bullet) that is contraindicated for magnetic resonance imaging (MRI) for safety reasons, or which may result in artefacts in medical imaging
Planning to undertake any major surgery in the next three years
Tobacco use
Alcohol or drug dependency
Taking medication that affects heart rate, body composition or bone mass (e.g., beta-blockers, glucocorticoids)
Having taken anti-resorptive therapy within the last three years
Having taken medication that affects appetite, metabolism, or weight within the past 6 months
Any of the following contraindications for following a very low energy/total meal replacement diet: lactose intolerance; following a strict vegan diet; or unwillingness to be randomized to one of the two diets
Donated whole blood within three months prior to commencement on the trial
Hepatic or renal impairments (which can be contraindications for following a very low energy/total meal replacement diet)

**Table 2:** Outcomes measured at each time point during the TEMPO Diet Trial.

Time (Months) from Start of Dietary Intervention	~0			~0.25	~1	~4			~6			~12		~24		~36	
Time (Weeks) from Start of Dietary Intervention	−2	−1	0	1	4	15	16	17 ^§^	25	26	29	51	52	103	104	155	156
**Body Composition**																	
Height			X														X
Weight (and Body Mass Index)			X	X	X		X	X		X			X		X		X
Bone																	
Bone Mineral Density and Bone Mineral Content																	
Hip (DXA)			X				X			X			X		X		X
Lumbar Spine (DXA)			X				X			X			X		X		X
Markers of Bone Turnover																	
Markers of Bone Formation																	
N-Terminal Propeptide of Type I Procollagen (P1NP) (Serum)			X				X			X			X		X		X
Osteocalcin (Serum)			X				X			X			X		X		X
Markers of Bone Resorption																	
C-Terminal Telopeptide of Type I Collagen (CTX) (Serum)			X				X			X			X		X		X
N-Terminal Telopeptide of Type I Collagen (NTX) (Urine)			X				X			X			X		X		X
Lean Tissues																	
Fat Free Mass (DXA)			X				X			X			X		X		X
Fat Free Mass (4-Compartment Model, Using): Bone Mineral Content (DXA) Body Volume (BodPod) Total Body Water (Deuterium Dilution)			X				X			X			X		X		X
Fat Free Mass (Using): Body Volume (BodPod) Total Body Water (Deuterium Dilution)			X	X	X		X	X		X			X		X		X
Thigh Muscle Cross Sectional Area (MRI)		X				X			X			X			X		X
Muscle Strength (Hand Dynamometry)			X				X	X		X			X		X		X
Fat Mass and Distribution																	
Waist Circumference			X	X	X		X	X		X			X		X		X
Hip Circumference			X	X	X		X	X		X			X		X		X
Ratio of Waist to Hip Circumference			X	X	X		X	X		X			X		X		X
Fat Mass (DXA)			X				X			X			X		X		X
Fat Mass (4-Compartment Model)			X				X			X			X		X		X
Fat Mass (Body Volume and Total Body Water)			X	X	X		X	X		X			X		X		X
Abdominal Fat (MRI)		X				X			X			X			X		X
Intrahepatic Lipid (^1^H-MRS)		X				X			X			X			X		X
Thigh Fat (MRI)		X				X			X			X			X		X
**Cardiometabolic Health (in Addition to Waist Circumference)**																	
Blood Pressure			X				X			X			X		X		X
Circulating Markers of Cardiometabolic Health																	
Glucose (Serum)			X				X			X			X		X		X
Glycosylated Hemoglobin (Whole Venous Blood)			X				X			X			X		X		X
Insulin (Serum)			X				X			X			X		X		X
Triglycerides (Serum)			X				X			X			X		X		X
Cholesterols (Serum)			X				X			X			X		X		X
Vascular Function and Structure																	
Flow Mediated Dilatation (FMD)			X				X			X			X		X		X
Carotid Intima Media Thickness (cIMT)			X							X			X		X		X
**Eating Disorder Behaviors/Psychology**																	
Eating Disorders Examination	X										X	X		X		X	
Eating Disorders Examination Questionnaire †	X				X		X			X	X	X		X		X	
Loss of Control over Eating Scale †	X				X		X			X	X	X		X		X	
Three Factor Eating Questionnaire	X										X	X		X		X	
Dutch Eating Behavior Questionnaire	X										X	X		X		X	
Mini International Neuropsychiatric Interview (MINI)																	
All Questions	X																
Lifetime History of Eating Disorder Questions	X																
Eating Disorder Questions Only												X		X		X	
General Mental Health																	
Rosenberg Self-Esteem Scale	X										X	X		X		X	
36-Item Short Form Survey (SF-36)	X										X	X		X		X	
Depression, Anxiety and Stress Scale (DASS-21)	X			X	X		X	X		X		X		X		X	
Mindfulness and Personality																	
Langer Mindfulness Scale	X																
Five Facet Mindfulness Questionnaire	X																
Big Five Inventory			X														
**Adaptive Responses to Energy Restriction**																	
Energy Intake, and Factors Influencing it																	
Food Diary			X	X ^⌘^	X ^⌘^		X	X		X			X		X		X
Drive to Eat																	
General Food Craving Questionnaire—State	X			X	X		X	X		X		X		X		X	
Eating Self-Efficacy Scale			X		X		X			X			X		X		X
Subjective Drive to Eat (Visual Analogue Scales)—Fasting and Postprandial			X	X	X		X	X		X			X		X		X
Appetite-Regulating Hormones																	
Ghrelin (Plasma)—Fasting and Postprandial			X	X	X		X	X		X			X		X		X
Peptide YY (Plasma)—Fasting and Postprandial			X	X	X		X	X		X			X		X		X
Leptin (Plasma)			X	X	X		X	X		X			X		X		X
Ketones																	
β-Hydroxybutyrate (Whole Venous Blood)			X	X	X		X	X		X			X		X		X
Acetoacetic Acid (Urine)			X	X	X		X	X		X			X		X		X
Physical Activity																	
Self-Efficacy to Regulate Exercise			X		X		X			X			X		X		X
Pedometer Step Count			X	X	X		X	X		X			X		X		X
Physical Activity Diary			X	X	X		X	X		X			X		X		X
Accelerometry			X	X	X		X	X		X			X		X		X
Sleep (in Addition to Accelerometry)																	
Sleep Diary			X	X	X		X	X		X			X		X		X
Epsworth Sleepiness Scale			X		X		X			X			X		X		X
Pittsburgh Sleep Quality Index			X		X		X			X			X		X		X
Energy Expenditure																	
Resting Energy Expenditure (Indirect Calorimetry)			X	X	X		X	X		X			X		X		X
Body Temperature			X	X	X		X	X		X			X		X		X
Neuroendocrine Status																	
Hypothalamo-Pituitary-Adrenal Axis																	
Cortisol (Serum)			X				X	X		X			X		X		X
Adrenocorticotropic Hormone (Plasma)			X				X	X		X			X		X		X
Hypothalamo-Pituitary-Thyroid Axis																	
Free Triiodothyronine or 3,3′,5-Triiodothyronine (T3) (Serum)			X				X	X		X			X		X		X
Reverse T3 (Serum)			X				X	X		X			X		X		X
Free Thyroxine or 3,5,3′,5′-Tetraiodothyronine (T4) (Serum)			X				X	X		X			X		X		X
Thyroid Stimulating Hormone (Serum)			X				X	X		X			X		X		X
Hypothalamo-Pituitary-Gonadotropic Axis																	
Estradiol (Serum)			X				X	X		X			X		X		X
Sex Hormone Binding Globulin (Serum)			X				X	X		X			X		X		X
Hypothalamo-Pituitary-Somatotropic Axis																	
Insulin-Like Growth Factor-1 (Serum)			X				X	X		X			X		X		X
Insulin-Like Growth Factor Binding Proteins (Serum)			X				X	X		X			X		X		X
Miscellaneous																	
Energy Homeostasis Questionnaire	X																
**General**																	
Preferred Intervention			X														
Diet Side Effect Questionnaire				X	X												

§ Only measured in participants in the FAST intervention. *⌘* While food diary data was collected at 8–9 time points, the food diaries served the dual purpose of providing data for analysis, as well as assisting with adherence to the prescribed dietary intervention. † The Eating Disorders Examination Questionnaire and the Loss of Control Over Eating Scale were additionally collected at 8, 12 and 20 weeks. All samples of blood and urine are collected in the fasting state unless otherwise stated, and except for the urine sample taken after deuterium administration for determination of total body water. All pedometer and accelerometer measures, as well as food, activity, and sleep diaries, were commenced a week prior to the measurement time point. DXA, Dual-energy X-ray absorptiometry; ^1^H-MRS, proton magnetic resonance spectroscopy; MRI, magnetic resonance imaging.
